# Anti-Colon Cancer Activity of Novel Peptides Isolated from In Vitro Digestion of Quinoa Protein in Caco-2 Cells

**DOI:** 10.3390/foods11020194

**Published:** 2022-01-12

**Authors:** Xin Fan, Huimin Guo, Cong Teng, Biao Zhang, Christophe Blecker, Guixing Ren

**Affiliations:** 1 Institute of Crop Science, Chinese Academy of Agricultural Sciences, No. 80 South Xueyuan Road, Haidian District, Beijing 100081, China; fx_926@163.com (X.F.); guohuimin0208@sina.com (H.G.); 82101172124@caas.cn (C.T.); 2 Gembloux Agro-Bio Tech, University of Liège, 5030 Gembloux, Belgium; christophe.blecker@uliege.be; 3 College of Pharmacy and Biological Engineering, Chengdu University, No. 1 Shilling Road, Chenglo Avenue, Longquan District, Chengdu 610106, China; zb17863201182@outlook.com

**Keywords:** hydrolysate, antiproliferative activity, HDAC1, inhibitory activity, molecular docking

## Abstract

Quinoa peptides are the bioactive components obtained from quinoa protein digestion, which have been proved to possess various biological activities. However, there are few studies on the anticancer activity of quinoa peptides, and the mechanism has not been clarified. In this study, the novel quinoa peptides were obtained from quinoa protein hydrolysate and identified by liquid chromatography–tandem mass spectrometry (LC–MS/MS). The anticancer activity of these peptides was predicted by PeptideRanker and evaluated using an antiproliferative assay in colon cancer Caco-2 cells. Combined with the result of histone deacetylase 1 (HDAC1) inhibitory activity assay, the highly anticancer activity peptides FHPFPR, NWFPLPR, and HYNPYFPG were screened and further investigated. Molecular docking was used to analyze the binding site between peptides and HDAC1, and results showed that three peptides were bound in the active pocket of HDAC1. Moreover, real-time quantitative polymerase chain reaction (RT-qPCR), and Western blot showed that the expression of HDAC1, NFκB, IL-6, IL-8, Bcl-2 was significantly decreased, whereas caspase3 expression showed a remarkable evaluation. In conclusion, quinoa peptides may have the potential to protect against cancer development by inhibiting HDAC1 activity and regulating the expression of the cancer-related genes, which indicates that these peptides could be explored as functional foods to alleviate colon cancer.

## 1. Introduction

The gastrointestinal tract is the main place for food digestion and nutrition absorption, and it is the organ that forms the protective barriers, including the mucosal gel layer, pH modulation, and the gut-associated lymphoid tissue, to the external environment [1,2]. In recent years, unhealthy eating habits and diet structure have led to an increasing trend of CRC incidence rate, showing a younger trend [3]. Thus, the interaction between foods and the digestive tract plays an important role in gastrointestinal health. Recent studies have proved the protective role of milk and whole grain on colorectal cancer, and plant-derived compounds possess the ability to inhibit malignant cell proliferation [4,5]. Because of their health-promoting benefits and no side effects, many plant-derived peptides are extensively applied in functional foods and nutraceuticals [6].

Quinoa (*Chenopodium quinoa* willd.) is a pseudocereal with higher nutritional properties due to dietary fiber, vitamins, minerals, and especially its larger amount of essential amino acids [7]. Recently, quinoa proteins and peptides have been demonstrated to possess a variety of beneficial effects, including antioxidant activity, immunometabolic effect, and antihypertensive effect [8,9,10,11]. Although quinoa protein has exhibited antiproliferative activity in human colorectal cancer cell lines after simulated gastrointestinal digestion [9], it is not clear which protein fragments or peptides are more effective and what their mechanism of action is in cancer inhibition.

Histone acetylation and deacetylation are important for the regulation of gene expression [12]. The family of HDACs includes 18 members, and class I HDACs (HDAC 1, 2, 3, and 8) is considered to be the most important ones [13]. Recent studies suggest that HDAC1 has been identified as a therapeutic target for cancer treatment [12]. For example, HDAC1 expression was increased in colorectal cancer and gastric cancer [14,15]. In contrast, HDAC1 knockdown causes cell cycle arrest, reduces cancer cell viability, and induces cancer cell apoptosis [16]. Moreover, pharmacological inhibitors of class I HDAC activity (HDACi) are potent inducers of growth arrest, differentiation, and apoptosis of colon cancer cells in vitro and in vivo [17]. Except for drug inhibitors, plant-derived peptides have also been shown to be effective inhibitors for the regulation of acetylation and deacetylation. Among them, soybean peptide lunasin has been widely studied as a chemopreventive agent with potential anti-cancer effects [18]. In colon cancer cells, lunasin can exert the anti-cancer activity through the regulation of histone acetylation and deacetylation, and the 50% inhibition of lunasin was found to be 61.7 µM [19,20]. In addition, the underlying molecular mechanisms of HDAC were also investigated. In breast cancer cells, HDAC1 can induce proliferation through the upregulation of Snail/IL-8 signals, and IL-8 can suppress the apoptosis of breast cancer MCF-7 cells by down-regulating caspase-3 and up-regulating Bcl-2 [21]. Meanwhile, IL-6 was also important as IL-8 for the progression of breast cancer cells, and the expression and release of IL-6 and IL-8 were regulated via MAPK and NF-κB mediated [22,23]. Moreover, HDAC1 has been demonstrated to promote cancer progression by activating HIF1α/VEGFA, ROS/TNF-α, and c-Myc/miR-34a signaling pathways [24,25,26]. However, there was no research about the quinoa derived peptides as HDAC1 inhibitors in colon cancer.

The purpose of this study was to assess the capability of quinoa peptides released during the simulated gastrointestinal digestion to affect the viability of human colon cancer Caco-2 cells, and screen peptides with HDAC1 inhibitory activity. The structure-activity relationship of these peptides was explored by molecular docking. Furthermore, RT-qPCR and Western blot were assayed to investigate the possible mechanism of quinoa peptides in alleviating colon cancer.

## 2. Materials and Methods

### 2.1. Materials and Reagents

Quinoa seed (*Mengli-I*) was purchased from the Inner Mongolia Yiji Biotechnology Company (Ulanqab, China). Pepsin from porcine gastric mucosa and pancreatin from porcine pancreas were from Sigma-Aldrich (St. Louis, MO, USA). Hydrochloric acid (HCL), sodium hydroxide (NaOH) and *n*-hexane were acquired from Sinopharm Chemical Reagent Co. Ltd. (Shanghai, China). The VivaFlow 200 tangential flow ultrafiltration system with a molecular weight cut-off (MWCO) of 5 kDa (Sartorius Stedim Biotech, Goettingen, Germany) was used in the ultrafiltration. The primers were synthesized by Sangon Biotech Corporation (Shanghai, China) and the primers used in this work are shown in Table S1. Specific primary antibodies against GAPDH, HDAC1, NFκB, IL-6, IL-8, Bcl-2, caspase3 were purchased from Bioss Inc. (Beijing, China).

### 2.2. Preparation of Quinoa Protein Concentrate, Quinoa Protein Hydrolysate and Quinoa Peptides

The quinoa protein concentrate (QP) was prepared following the protocol of Guo et al. with slight modifications [11]. Quinoa seeds were washed five times with distilled water to remove saponins, and dried in a 50 °C drying oven. The dried seeds were ground to flour by using FOSS CT293 Cyclotec (Foss Tecatur AB, Hillerød, Denmark), sifted through a 60-mesh sieve, and defatted with n-hexane. The defatted quinoa flour was suspended in water (1:10, *w*/*v*), and its pH was adjusted to 8.0 with 2 M NaOH. The suspension was stirred for 1 h and centrifuged at 6000× *g* for 30 min at room temperature. Adjusting the pH of the supernatant to 4.0 with 2 M HCl. The supernatant stabilized for 1 h at 4 °C and was then centrifuged for 30 min at 6000× *g*. The precipitate was freeze-dried and stored at 4 °C for the next experiment.

The quinoa protein hydrolysate (QPH) was prepared following the previous study [19]. Briefly, QP was dissolved in deionized water (1:100, g/mL) and adjusted the pH of QPH solution to pH 2.0 with addition of 1M HCl. Then the solution was hydrolyzed by trypsin (5%, *w*/*w*) for 1 h in a 37 °C water bath shaker, stopping the reaction by adjusting the pH at 7.0 with 1 M NaOH. Simulated intestinal fluid containing pancreatin (5%, *w*/*w*) was mixed with hydrolysate in the gastric phase for 1 h at 37 °C in a water bath. The reaction was stopped in a boiling water bath for 5 min. QPH digest was cooled to room temperature and collected after centrifugation. The suspension was frozen and stored at −20 °C. The control sample without enzyme was used in the same condition.

QPH digest was filtered through a 5 kDa ultrafiltration membrane. Fractions MW < 5 kDa and MW > 5 kDa were obtained and frozen dried and kept at −20 °C until further analysis. The fraction with high antiproliferative activity was further identified using electrospray ionization quadrupole time-of-flight mass spectrometry as described in our previous study [11].

### 2.3. Peptide Screening and Synthesis

The potential peptide candidates were searched against the BIOPEP database (http://www.uwm.edu.pl/biochemia, accessed on 5 May 2021). The potential biological activity of these identified peptides was predicted using the scores calculated in PeptideRanker (http://distilldeep.ucd.ie/PeptideRanker/, accessed on 5 May 2021) and the theoretical bioactivity was scored from 0 to 1 so that the higher value means a higher probability of being bioactive. The toxicity of all these peptides was scored in ToxinPred (http://crdd.osdd.net/raghava/toxinpred/, accessed on 5 May 2021).

The predicted potential bioactivity peptides were synthesized through the conventional Fmoc solid-phase synthesis method at Sangon Biotech Co., Ltd. (Shanghai, China). The purity of the peptide was verified by high performance liquid chromatography (HPLC). Molecular masses were confirmed using liquid chromatography–tandem mass spectrometry (LC–MS/MS).

### 2.4. Cell Proliferation Assay

The human CRC cell line Caco-2 was obtained from American Type Cell Collection (ATCC, Manassas, VA, USA) and cultured in MEM medium supplemented with 20% FBS, 1% penicillin, and 1% streptomycin. Cells were maintained at 37 °C in a humidified incubator containing 5% CO_2_ and 95% air. The medium was changed every 24 h. All cells were assayed within 5–15 passages. Utilizing the CCK-8 kit (Biorigin, Beijing, China), the toxicity test was first performed. Caco-2 cells (100 µL) were incubated in the 96-well plate (1 × 10^5^ cells/well) for 24 h, then the original medium was replaced with fresh medium containing different concentrations of QP (1–8 g/L), QPH (1–8 g/L), fraction MW < 5 kDa (1–8 g/L), fraction MW > 5 kDa (1–8 g/L), and selected peptides ranging from 0 to 2 g/L. After 24 h co-cultivation, the medium was discarded and washed with PBS 2 times, and a fresh medium with 10% CCK-8 reagent was added to each cell. Another 1 h later, the absorbance (OD value) was determined by an enzyme-labeling instrument at a wavelength of 450 nm. Experiments were performed in triplicate with at least three replicates per concentration, and results were expressed as percentage of negative control (non-treated Caco-2 cells).

For the proliferation experiments, Caco-2 cells were seeded into 96-well plates at a density of 2.5 × 10^4^ per well for 24 h, the incubation was continued for 72 h. After that, the subsequent washing and CCK-8 reaction process were the same as performed in the toxicity test. Finally, the absorbance was also detected at 450 nm. Analyses were performed in triplicate with at least three replicates per concentration, and results were also shown as the percentage of non-treated Caco-2 cells.

### 2.5. HDAC1 Inhibitory Activity Assay

HDAC1 activity was determined using the HDAC1 inhibitor screening assay kit (Abnova, Taipei, Taiwan). The assay was performed according to the manufacture instruction manual. Briefly, the sample wells were added 140 µL assay buffer, 10 µL diluted HDAC1, and 10 µL peptide samples (0.05–2 g/L). The trichostatin A was set as the positive control, and the sample buffer was set as the negative control. We initiated the reactions by adding 10 µL of HDAC substrate to all the wells being used. Cover the plate with the plate cover and incubate on a shaker for 30 min at 37 °C. Remove the plate cover and add 40 µL of developer. Cover the plate with the plate cover and incubate for 15 min at room temperature. Remove the plate cover and read the fluorescence using an excitation wavelength of 340–360 nm and an emission wavelength of 440–465 nm. IC_50_ values were calculated using GraphPad Prism 8 software. Analyses were performed in triplicate with at least three replicates per concentration.

### 2.6. Molecular Docking

The crystal structure of HDAC1 was obtained from the Protein Data Bank (PDB ID: 4BKX). Water, sulfate and acetate molecules were removed from the system. Meanwhile, structural zinc ion was left because of its importance in the catalytic activity and conserved position of the crystal structure of HDAC1. The structure of studied peptides was constructed using UCSF Chimera 1.13.1. Molecular docking simulations were performed using MGL Tools 1.5.6 and AutoDock Tools 4.2 [27], and the grid box was centered on Zn^2+^. The best docking models of peptides in the active pocket of HDAC1 were displayed according to the binding energy value. Finally, the binding sites were analyzed by using PyMOL 1.5.0.3.

### 2.7. Quantitive Real Time-Polymerase Chain Reaction (RT-qPCR)

After co-cultivation with different concentrations of selected peptides, total RNA of Caco-2 cells was extracted using TRIzol Reagent (Life Technologies, Carlsbad, CA, USA) according to the manufacturer’s protocol. Total RNA was treated with DNase I and reverse-transcribed into cDNA using the cDNA Synthesis SuperMix kit (TransGen Biotech, Beijing, China). The mRNA expressions were then quantified by RT-qPCR using the TransStart Top Green qPCR Supermix kit (TransGen Biotech, Beijing, China) and the Applied Biosystems 7500 real-time PCR systems (Applied Biosystems, Foster City, CA, USA). Analyses were performed in triplicate with at least three replicates per sample, and gene expression was normalized to the geometric mean of reference genes (GAPDH) using the 2^−ΔΔCt^ method.

### 2.8. Western Blot Analysis

Total proteins were extracted using mammalian protein extraction kit (CWBIO, Beijing, China). After being treated with different concentrations of selected peptides, Caco-2 cells were washed three times with PBS and incubated with lysis buffer on ice for 20 min. Subsequently, the cell lysate was centrifuged at 12,000× *g* at 4 °C for 20 min to collect the supernatant. The protein samples for each treatment were loaded and separated in 12% sodium dodecyl sulfate-polyacrylamide gel. Then, all samples were transferred to an Immun-Blot PVDF membrane (0.22 um) using Bio-Rad trans-blot apparatus (Bio-Rad, Cambridge, MA, USA). When the transfer was achieved, the membrane was blocked in blocking buffer at room temperature. After 1 h of blocking, the membrane was incubated with primary antibody at 4 °C for overnight, followed by incubation with a secondary antibody (goat anti-rabbit IgG-HRP) for 1 h at room temperature. After hybridization, the membrane was incubated in immobilon ECL ultra Western HRP substrate (Millipore Corporation, MA, USA) for 20 min. The specific band for the target protein was detected by Tanon 5200 (Tanon Corporation, Shanghai, China). The bands were quantified using ImageJ, and the intensities of the bands were normalized to loading control (GAPDH). Experiments were repeated at least four times per sample.

### 2.9. Statistical Analysis

All data are expressed as the means ± standard deviation (SD). Differences among groups were determined by one-way ANOVA analysis and Duncan’s multiple range tests with SPSS software (IBM, New York, NY, USA). Significant differences were expressed at *p* < 0.05 and *p* < 0.01.

## 3. Results

### 3.1. Antiproliferative Activity of QPH against Caco-2 Cells

The quinoa protein yield and purity were detected according to the previous method, the results were 6.85% and 86.02%, respectively. The hydrolysis degree of QPH was examined by Adler–Nissen’s method and the result was 20.46%.

**Figure 1:**
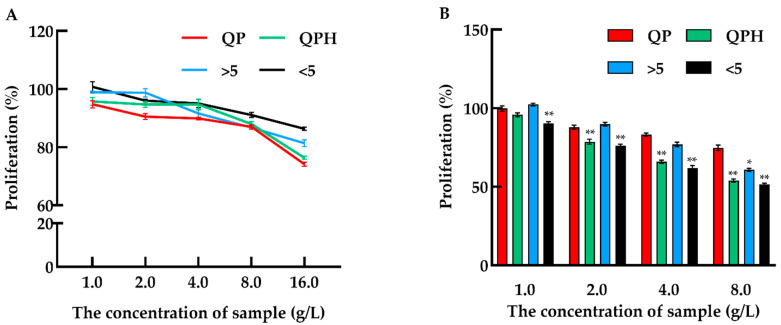
Cytotoxic effect and anti-proliferative activity of different fractions from quinoa protein on Caco-2 cells. (**A**) Dose dependent effect of different quinoa protein fractions on Caco-2 cells after 24 h treatment; (**B**) the cell proliferation rate of Caco-2 cells under gradient concentration of quinoa protein fractions after 72 h treatment. QP: quinoa protein concentrate, QPH: quinoa protein hydrolysate, >5 kDa: fractions MW > 5 kDa, <5 kDa: fractions MW < 5 kDa. * *p* < 0.05 and ** *p* < 0.01 indicate significant and highly significant differences between the QP and other treatments, respectively.

In this study, the toxicity test of QPH was performed in Caco-2 cells. The number of viable cells was calculated by a CCK-8 assay after being incubated with different concentrations of samples for 24 h. As shown in Figure 1A, no toxicity was observed over four groups at concentrations ranging from 1 to 8 g/L. When the concentrations of samples were up to 16 g/L, four groups all showed slight toxicity. The antiproliferative activity was assessed using a CCK-8 assay. The result was shown in Figure 1B, the proliferation of Caco-2 cells was gradually inhibited with the increasing sample concentration (1–8 g/L) after 72 h incubation with samples. Meanwhile, the QPH and fraction < 5 kDa have higher inhibitory activities than QP and fraction > 5 kDa, and the inhibition rate under QPH and fraction < 5 kDa treatment can reach 51.45 and 53.93%, respectively, at a concentration of 8 g/L.

### 3.2. In Silico Analysis and Antiproliferative Activity of Quinoa Peptides

Based on the results of the previous step, fraction < 5 kDa possessed stronger antiproliferative activity. Using ESI-Q-TOF–MS/MS to detect the quinoa peptides of fractions < 5 kDa, the identification and in silico analysis results are listed in Table 1, and the MS spectrum are shown in Figure S1. The bioactivity of identified peptides was predicted using the PeptideRanker program, and thirteen quinoa peptides were predicted to have high bioactivity with predicted scores over 0.7. The prediction results of ToxicPred showed that none of them were considered to be toxic. Among these peptides, some of them share similar amino acid sequences. For example, FHPFPR was measured as a part of PNFHPFPR, SLPNFHPFPR and NSLSLPNFHPFPR. Similarly, SVENWFPLPR and INNIFRPF contained sequence NWFPLPR and NIFRPF, respectively. Nevertheless, according to the PeptideRanker score results, the shorter peptides, including FHPFPR, NWFPLPR and NIFRPF, have higher activity.

Subsequently, a CCK-8 assay was conducted to characterize which peptides are mostly responsible for inhibiting cancer cell proliferation. The results were listed in Table 2. In a certain range, the inhibition rate increased with increased peptides concentrations. However, there was no significant change was observed while the concentration of peptides was up to 2 g/L. Among these peptides, the peptides FHPFPR, PNFHPFPR, NWFPLPR and HYNPYFPGGA had more than 30% inhibition at a concentration of 1 g/L.

### 3.3. HDAC1 Inhibitory Activity of Quinoa Peptides

**Figure 2:**
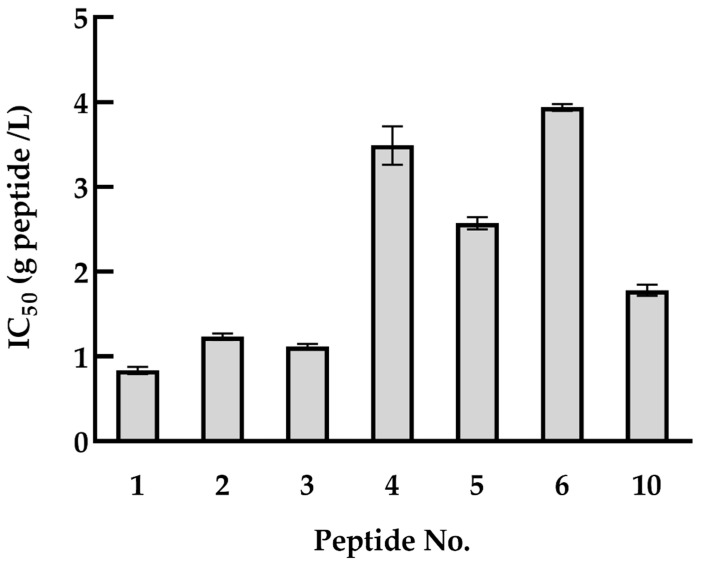
HDAC1 inhibitory activities of quinoa peptides obtained from QPH fraction < 5 kDa. 1: FHPFPR, 2: NWFPLPR, 3: PNFHPFPR, 4: NIFRPF, 5: SLPNFHPFPR, 6: INNIFRPF, 10: HYNPYFPGGA. HDAC1: histone deacetylase 1, QPH: quinoa protein hydrolysate.

To measure the effect of the identified quinoa peptides on HDAC1 activity, an HDAC1 inhibitory activity assay was conducted. For these peptides, half of them can reach to IC50 value, including FHPFPR, PNFHPFPR, NWFPLPR, HYNPYFPGGA, NIFRPF, SLPNFHPFPR and INNIFRPF (Figure 2). The rest of the peptides failed to attain the IC50 value. Moreover, the shorter peptides have higher HDAC1 inhibitory activity, as predicted in Section 3.2. Taken together with the result of cell proliferation, quinoa peptides FHPFPR, NWFPLPR and HYNPYFPG were used for the next experiments, and their HDAC1 inhibitory activity were performed with IC50 values of 0.87, 1.27, and 1.85 g/L, respectively.

**Figure 3:**
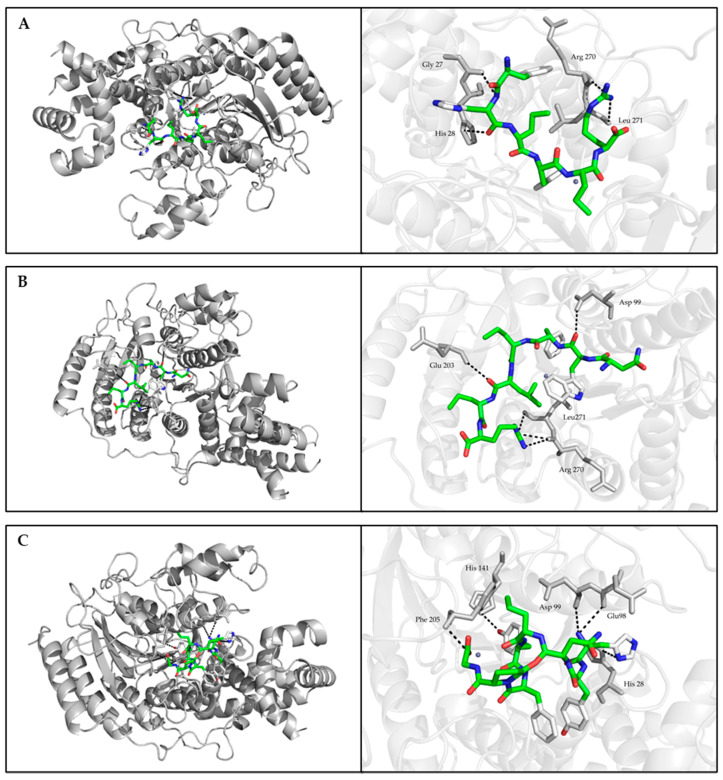
Best binding mode between HDAC1 and studied peptides retrieved from focused molecular docking. (**A**) FHPFPR, (**B**) NWFPLPR, (**C**) HYNPYFPGGA. The general overview was shown in the left panel. The interaction (block dotted lines) between the residues of HDAC1 (grey sticks) and peptides (green sticks) was zoomed in the right panel.

Molecular docking simulations of HDAC1-peptide were conducted using the AutoDock Vina program of AutoDock Tools 4.2. The best binding poses of FHPFPR, NWFPLPR, HYNPYFPGGA inhibit HDAC1 activity were shown in Figure 3, and the binding energy of these peptides were −6.56, −6.48, −6.41 kcal/mol.

### 3.4. The Effect of Quinoa Peptides on HDAC1-Induced Cancer Progression

**Figure 4:**
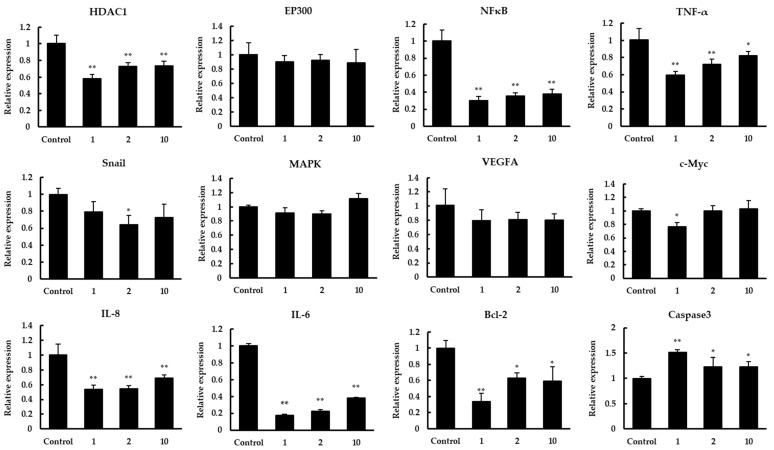
The effect of quinoa peptides 1, 2, 10 on the mRNA transcript levels of HDAC1, EP300, NFκB, TNF-α, Snail, MAPK, VEGFA, c-Myc, IL-8, IL-6, Bcl-2, and caspase3. The mRNA expression results are displayed as the fold increase of mRNA expression normalized to GAPDH and shown in mean ± SD. * *p* < 0.05 and ** *p* < 0.01 versus control group.

Targeted inhibition of histone deacetylase (HDAC) is an effective anticancer therapy [19]. To study which regulation HDAC1 may participate in to inhibit the proliferation of colon cancer Caco-2 cells, qPCR and Western blot were carried out to detect related target genes. The results of mRNA expression were shown in Figure 4. After treatment with peptides 1, 2, 10 in Caco-2 cells, the HDAC1 expression was significantly suppressed, whereas the expression of EP300 was not significantly changed. Previous studies reported that two small soluble members of CXC chemokine family, IL-6 and IL-8, are critical to the development of cancer cells [22,28]. We detected the expression of these genes, and the results showed that there was a significant decrease in IL-6 and IL-8 expression after treatment. Compared to the control, the expression of IL-6 and IL-8 was downregulated to 0.18–0.38 and 0.54–0.69 fold, respectively. Further research suggested IL-8 can inhibit the apoptosis of human breast cancer cells via up-regulating Bcl-2 and down-regulating caspase-3 [21]. In our study, Bcl-2 expression was inhibited with the down-regulation of IL-8 while caspase3 expression was significantly decreased. In addition, NF-κB and MAPK were important signals responsible for the transcriptions of IL-8 and IL-6 [23]. Similarly, as the key transcription factor for epithelial–mesenchymal transition (EMT) of cancer cells, Snail can regulate the expression of IL-8 positively in cancer cells [29]. The expression results showed that NF-κB was significantly repressed after treatment while not MAPK, and there was a slight downregulation in the Snail expression. In previous studies, tumor-related genes TNF-α, VEGFA, and c-Myc were also regulated by HDAC1 [24,25,26]. We tested the effects of quinoa peptides on the expression of TNF-α, VEGFA, and c-Myc. The data showed that TNF-α expression fold change was downregulated to 0.60–0.82. However, there was a minor decrease in the expression of VEGFA and c-Myc.

**Figure 5:**
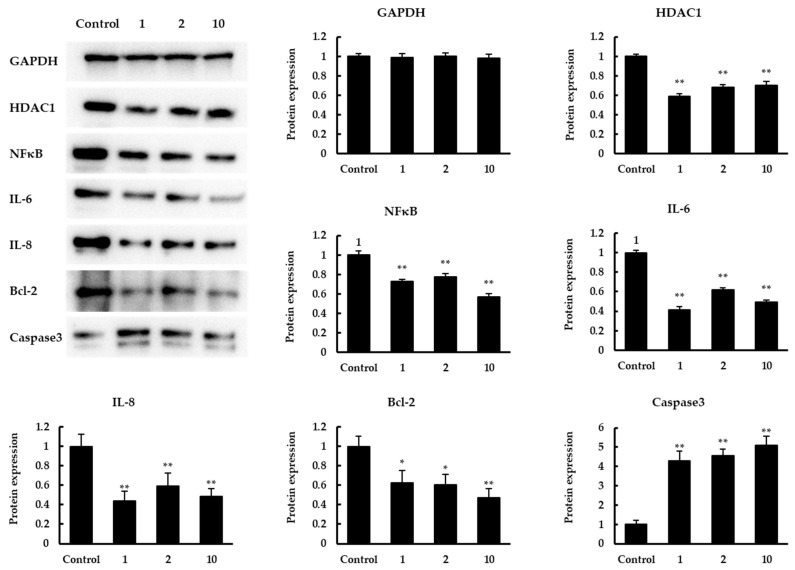
Western blot analysis. The effect of quinoa peptides 1, 2, 10 in protein levels of GAPDH, HDAC1, NFκB, IL-6, IL-8, Bcl-2, caspase3. GAPDH was set as the loading control. Expression was normalized to GAPDH expression and shown in mean ± SD. * *p* < 0.05 and ** *p* < 0.01 versus control group.

Subsequently, a Western blot assay was conducted to confirm the expression in protein levels of related genes with significant changes mentioned above (Figure 5). The results showed that the expression levels of HDAC1, IL-6, IL-8, NFκB, Bcl-2 were significantly increased. In contrast, there was a significant increase in caspase3 expression. The expression levels of all the above proteins were consistent with the results on the transcription level.

## 4. Discussion

Colon cancer is a common malignant tumor in the digestive system and is difficult to detect in the early stages. Many risk factors are affecting the occurrence and progress of colon cancer, including age, chronic alcohol, unhealthy diet, and so on. Although some synthetic drugs are currently utilized for the treatment of colon cancer, which is insufficient and may have side effects. Recently, dietary and microbial components are expected as therapeutic adjuvants to inhibit the occurrence of colon cancer [30]. Compared to other cereals, quinoa was rich in protein with excellent nutritional value, which can release bioactive peptides after enzymatic hydrolysis [9,31]. Moreover, quinoa protein and its derived peptides have shown certain antihypertensive activity and the greatest anti-cancer effects [9,11].

In this study, the antiproliferative activities of different samples were evaluated in colon cancer Caco-2 cells. Vilcacundo and co-workers have proved that sequential incubation with pepsin and pancreatin result in the complete degradation of quinoa protein while incubation with pepsin resulted in the partial hydrolysis of quinoa protein. They also demonstrated that the antiproliferative activity of gastroduodenal digests was significantly more increased than that exhibited by gastric digest [9]. We followed their digestion conditions to prepare QPH and found that QPH exhibited an increasing antiproliferative activity while QP did not show obvious anticancer ability in the concentration range from 0–4 g/L, which is consistent with a previous study [9]. This result suggests that more peptides of quinoa protein were released by pepsin and pancreatin after an in vitro simulation of digestion.

To investigate the effect of the molecular weight of quinoa peptides on the proliferation of Caco-2 cells, QPH was separated into two parts: fraction > 5 kDa and fraction < 5 kDa. Compared to fraction > 5 kDa, fraction < 5 kDa showed a dose-dependent proliferation inhibitory activity, indicating that low molecular weight quinoa peptides possess more effective antiproliferative activity than high molecular weight peptides. Jumeri et al. have reported the greater molecular mobility and diffusivity of low molecular weight peptides were identified to improve interactions with cancer cell components and enhance antiproliferative activity [32]. There have been several studies about the peptides derived from different crops as nutraceuticals in the prevention or the treatment of cancer. Kannan and co-workers found the fraction < 5 kDa of rice bran protein hydrolysates imparts a stronger anti-cancer activity against Caco-2 and HepG2 cells than fraction 5–10 kDa and fraction > 5 kDa [33]. Similar results were also observed in the study of González-Montoya et al., the fraction < 10 kDa of germinated soybean digests showed a higher ability to inhibit Caco-2 proliferation than the fraction > 10 kDa [34]. However, there was a difference in soybean digests, Rayaprolu and co-workers identified that fraction 10–50 kDa extracted from soybean meal protein was the most potent component inhibiting human liver and colon cells viability [35]. Additionally, the antiproliferative activity of quinoa peptides in human colon cancer cells has been measured in a recent study, and the fraction > 5 kDa exhibited more suppressive activity than fraction < 5 kDa [9]. Although the hydrolysates were obtained from the same crop, the same molecular weight fractions may have different anti-cancer activities, which can be attributed to several factors including crop cultivars, environment areas, enzymatic hydrolysis conditions, amino acid composition, and peptide hydrophobicity.

To identify the potential peptides responsible for the antiproliferative activity, a fraction < 5 kDa was analyzed by LC–MS/MS. The bioactivity of identified peptides was predicted using PeptideRanker and the peptides with predicted scores over 0.7 were listed in Table 1. Shorter sequences had higher inhibitory activity compared to the other similar sequence, which is consistent with the Jumeri’ conclusion mentioned above. The result of the antiproliferation experiment showed that quinoa peptides FHPFPR, NWFPLPR, and HYNPYFPG were more active in inhibiting the proliferation of colon cancer cells. It has been previously reported that antioxidant peptides have the potential to prevent and treat reactive oxygen species-related diseases, especially some forms of cancer [36]. These antioxidant peptides can be used as anticancer compounds to reduce genetic alterations such as mutation and chromosome rearrangement by inhibiting oxidative stress [32]. Furthermore, amino acids tyrosine (Y), tryptophan (W), methionine (M), cysteine (C), histidine (H), and phenylalanine (F) were considered as the main contributors to the radical-scavenging activities of peptides [37]. The quinoa peptides with MW < 5 kDa also have exhibited antihypertensive activity via inhibiting the activity of ACE, and the ACE inhibitors have been shown to be associated with the colorectal cancer risk in a duration-response manner [11,38]. In a recent study, the quinoa peptides containing the sequences PR and FP have been demonstrated to have ACE inhibitory activity [11]. Combined with previous studies and our experimental results, the quinoa peptides with antioxidant and antihypertensive activities played significant roles in alleviating colon cancer.

The molecular docking analysis further explained the possible inhibition mechanism. Structural analysis showed that the active site of HDAC1 protein crystal structure was a long tunnel that leads to the cavity of the catalytic machinery containing zinc ions. The tunnel is mainly hydrophobic and consists of amino acids His140, His141, Gly149, Asp176, His178, His179, Phe205, and Asp264 [39]. The HDAC1 inhibitors, suberoylanilide hydroxamic acid (SAHA), and the macrocyclic peptide FK228, were investigated in previous studies [39,40]. The re-docking complex of the inhibitor SAHA and HDAC1 showed that SAHA had hydrogen bonds with His178, Asp176, and hydrophobic interactions with His140, His141, and Tyr303 of HDAC1 [39]. Moreover, the docking conformations of FK228 in the binding pockets of HDAC1 proved that the hydrophobic interaction and hydrogen bonding of Glu91, Asp92 in the Loop2 and His 21, Pro22 in the Loop1 of HDAC1 were associated with HDAC1 activity [40]. In this study, the molecular docking result showed that HYNPYFPG had hydrogen bonds with Glu98, Asp99, His28, His141, and Phe205. The different positions of amino acid Glu, Asp, and His were due to the reconstruction of FK228 in HDAC1 that leads to the rearrangement of amino acid numbers. These binding sites between HYNPYFPG and HDAC1 were considered to inhibit the enzyme activity. Although FHPFPR and NWFPLPR were also bound in the active pocket of HDAC1, only His28 and Asp99 were reported to inhibit HADC1 activity. However, it should be mentioned that both FHPFPR and NWFPLPR displayed direct interactions with residues Arg270 and Leu271, which might be responsible for their HDAC1 inhibitory activity. Meanwhile, it has been demonstrated that the inhibitory peptides based on a non-competitive inhibition mechanism could not bind to the active residue of receptor-enzyme [41]. Thus, FHPFPR, NWFPLPR and HYNPYFPG have potential as the novel HDAC1 inhibitors.

Although quinoa peptides have been demonstrated to possess various bioactivities, the mechanisms by which these anti-cancer peptides exert their inhibitory effect in human colon cancer cells are not fully understood. Histone acetylation and deacetylation have been revealed as important factors for the regulation of cancer progression [42]. Studies have shown that abnormal histone deacetylation is associated with malignant tumors and HDACi can inhibit cancer progression through remodeling histone acetylation [43]. Until recently, the inhibitory effect of HDAC1 has been proved in different types of cancer cells, including breast cancer, gastric cancer, pancreatic cancer, non-small cell lung cancer, and colon cancer. In addition to the drugs with inhibitory effects, the soybean peptide lunasin can induce apoptosis in cancer cells by regulating histone acetylation. In this study, the mRNA expression of HDAC1 in quinoa peptides treatment group was decreased while EP300 displayed no significant effect. Similar to previous studies, the mRNA expression of IL-8, Bcl-2 was significantly inhibited, and caspase3 was up-regulated compared to the control group. Tang and co-workers demonstrated that HDAC1 can positively regulate the transcription and promoter activities of IL-8, while not IL-6, in breast cancer cells [21]. They suggested that NF-κB and MAPK signals were not involved in the regulation of IL-8. However, we found that the expression of NF-κB, IL-6 was also significantly repressed after treatment, which is supported by prior studies. It has been proved that HDACi vorinostat can suppress the activation of IL-8 promoter in Caco-2 cells via NF-κB-independent pathways [44]. Moreover, Choi et al. have indicated that vorinostat can inhibit the expression of IL-6 and IL-8 in peripheral blood mononuclear cells (PBMCs) from treated patients [45]. In our previous study, lunasin of transgenic wheat also had inhibitory effect on colon cancer cells by modulating the apoptosis pathway, and the expression of Bax and caspase3 was increased while Bcl-2 was decreased [46]. Furthermore, TNF-α, VEGFA, and c-Myc were considered to serve important roles in tumorigenesis and tumor development, which can be regulated by HDAC1 [24,25,26]. Among these genes, only TNF-α was significantly downregulated in this study, which was proved that mitochondrial reactive oxygen species (ROS) induces HDAC activation through c-Src signaling in LPS-stimulated cardiomyocytes, and HDAC activation and mitochondrial ROS promote LPS-stimulated TNF-α expression in cardiomyocytes [24]. One possible explanation for these discrepancies is that HDAC1 activity was inhibited by use of siRNAs to knockdown its expression. However, the quinoa peptides were not highly specific for inhibiting HDAC1. Therefore, our results suggested that the quinoa peptides inhibit cell proliferation by regulating various pathways in colon cancer Caco-2 cells.

## 5. Conclusions

This study revealed that QPH possesses the potential to inhibit the proliferation of colon cancer cells. The novel peptides, FHPFPR, NWFPLPR, HYNPYFPGGA, identified from QPH were also proved to exert a promising antiproliferative activity. Combined with the result of the HDAC1 inhibitory assay, molecular docking further showed the binding mode between peptides and HDAC1, and stated the presence of specific amino acids in the peptide sequence may contribute to the binding interaction. Moreover, the results of RT-qPCR and Western blot indicated that these peptides may serve their function by inhibiting HDAC1 activity and modulating cancer-related gene expression. However, the effects of quinoa peptides on colon cancer still need to be further investigated to study their molecular mechanism in related markers and signaling pathways, and to verify the anti-colon cancer activity in vivo.

## Figures and Tables

**Table 1:** Identification of quinoa peptides (fraction < 5 kDa) and their PeptideRanker score and toxicity.

No.	Peptide	Ion (*m*/*z*)	Calculated Mass	Peptides Score	Toxicity
1	FHPFPR	799.90	799.92	0.9745	Non
2	NWFPLPR	929.00	929.08	0.9722	Non
3	PNFHPFPR	1011.15	1011.14	0.9631	Non
4	NIFRPF	793.00	792.92	0.9402	Non
5	SLPNFHPFPR	1211.40	1211.38	0.9388	Non
6	INNIFRPF	1020.20	1020.19	0.8666	Non
7	SVENWFPLPR	1244.50	1244.40	0.8594	Non
8	NSLSLPNFHPFPR	1525.80	1525.72	0.8383	Non
9	NSWGPNWGDHG	1226.10	1226.22	0.8118	Non
10	HYNPYFPGGA	1122.20	1122.19	0.8021	Non
11	FGGGTLGHPW	1028.10	1028.12	0.7855	Non
12	GLESPNYPWPH	1296.40	1296.39	0.7791	Non
13	HGSLGFLPR	983.10	983.13	0.7758	Non

**Table 2:** Dose-dependent effects of quinoa peptides released from gastrointestinal digestion on cell viability.

Peptide	Viable Cells (%) at Different Concentrations (g/L)				
	0	1 × 10^−1^	5 × 10^−1^	1	2
FHPFPR	99.96 ± 1.53	90.04 ± 1.26 ^e^	75.46 ± 0.86 ^f^	51.41 ± 0.80 ^k^	50.82 ± 0.76 ^j^
NWFPLPR	101.01 ± 1.12	92.86 ± 1.10 ^d^	79.99 ± 0.86 ^e^	64.02 ± 1.39 ^j^	63.18 ± 0.83 ^i^
PNFHPFPR	100.97 ± 2.29	90.46 ± 1.43 ^e^	77.26 ± 1.65 ^f^	65.32 ± 1.45 ^j^	61.67 ± 1.64 ^i^
NIFRPF	100.75 ± 1.69	100.10 ± 1.03 ^a^	85.11 ± 0.66 ^d^	70.29 ± 0.56 ^h^	70.55 ± 2.01 ^g^
SLPNFHPFPR	99.55 ± 0.95	97.79 ± 1.14 ^bc^	87.21 ± 0.32 ^d^	78.51 ± 0.26 ^g^	78.00 ± 0.80 ^f^
INNIFRPF	100.40 ± 0.39	100.40 ± 1.29 ^a^	92.05 ± 1.07 ^c^	80.44 ± 0.68 ^f^	80.61 ± 0.41 ^e^
SVENWFPLPR	100.42 ± 0.75	99.43 ± 1.39 ^ab^	95.50 ± 3.60 ^b^	92.14 ± 0.69 ^c^	91.04 ± 1.53 ^c^
NSLSLPNFHPFPR	99.21 ± 1.60	100.79 ± 0.52 ^a^	92.78 ± 2.20 ^c^	86.55 ± 0.58 ^e^	83.56 ± 0.71 ^d^
NSWGPNWGDHG	101.06 ± 1.21	98.87 ± 0.60 ^abc^	98.60 ± 2.28 ^a^	99.58 ± 1.08 ^a^	97.92 ± 1.41 ^a^
HYNPYFPGGA	101.54 ± 0.74	93.69 ± 0.68 ^d^	82.34 ± 1.36 ^e^	67.44 ± 1.26 ^i^	67.10 ± 1.87 ^h^
FGGGTLGHPW	98.81 ± 1.76	97.09 ± 0.74 ^c^	91.65 ± 0.43 ^c^	90.41 ± 0.69 ^d^	89.72 ± 0.60 ^c^
GLESPNYPWPH	100.33 ± 1.34	99.75 ± 1.06 ^a^	99.32 ± 1.05 ^a^	97.40 ± 0.73 ^b^	97.70 ± 0.33 ^ab^
HGSLGFLPR	100.26 ± 1.66	97.35 ± 1.03 ^c^	96.91 ± 0.77 ^ab^	96.81 ± 0.88 ^b^	95.80 ± 0.62 ^b^

Results are shown as mean ± standard deviation (SD). Values not sharing the same small letter in a column indicate significant changes between samples, by Fisher’s test (*p <* 0.05).

## Data Availability

All data are reported in this manuscript.

## Supplementary Materials

The following are available online at https://www.mdpi.com/article/10.3390/foods11020194/s1, Figure S1: MS/MS spectrum of the novel quinoa peptides from fraction < 5 kDa, Table S1: Primers used in this study.
