# Supplementary material for: Anti-Colon Cancer Activity of Novel Peptides Isolated from In Vitro Digestion of Quinoa Protein in Caco-2 Cells
Source: Foods. 2022 Jan 12;11(2):194. doi: 10.3390/foods11020194 (PMC8774364; doi:10.3390/foods11020194)
Supplement: Supplementary file 1 [file foods-11-00194-s001.zip › foods-1444630-supplementary.pdf]

Table S1. Primers used in this study.

Primer name	Gene	Sequence (5′ - 3′)
GAPDH - F	GAPDH	GTATCGTGGAAGGACTCATGAC
GAPDH - R	GAPDH	ACCACCTTCTTGATGTCATCAT
HDAC1 - F	HDAC1	ATCCGCATGACTCATAATTTGC
HDAC1 - R	HDAC1	GGATGGAGCGCAAGAATTTAAT
EP300 - QF	EP300	GTTCCTTCCTCAGACTCAGTTC
EP300 - QR	EP300	CATTATAGGAGAGTTCACCGGG
NFκB - F	NFκB	GGCGAGAGGAGCACAGATAC
NFκB - R	NFκB	CGGCAGTCCTTTCCTACAAG
TNF-α- F	TNF-α	TAGCCCATGTTGTAGCAAACCC
TNF-α- R	TNF-α	GGACCTGGGAGTAGATGAGGT
Snail - F	Snail	TTTACCTTCCAGCAGCCCTA
Snail - R	Snail	GGACAGAGTCCCAGATGAGC
MAPK - F	MAPK	TGCACATGCCTACTTTGCTC
MAPK - R	MAPK	AGGTCAGGCTTTTCCACTCA
VEGFA - F	VEGFA	GCAGAATCATCACGAAGTGGT
VEGFA - R	VEGFA	CCAGGGTCTCGATTGGATGG
c-Myc - F	c-Myc	AGCGACTCTGAGGAGGAACA
c-Myc - R	c-Myc	CTCTGACCTTTTGCCAGGAG
IL-8 - F	IL-8	GTGCAGTTTTGCCAAGGAGT
IL-8 - R	IL-8	AAATTTGGGGTGGAAAGGTT
IL-6 - F	IL-6	AGACAGCCACTCACCTCTTCA
IL-6 - R	IL-6	TTCTGCCAGTGCCTCTTTGCT
Bcl-2 - F	Bcl-2	TGGGATTCCTGCGGATTGAC
Bcl-2 - R	Bcl-2	GTCTACTTCCTCTGTGATGTTGT
Caspase3 - F	Caspase3	GGTTCATCCAGTCGCTTTGT
Caspase3 - R	Caspase3	CGGTTAACCCGGGTAAGAAT

Figure S1. MS/MS spectrum of the novel quinoa peptides from fraction < 5 kDa.

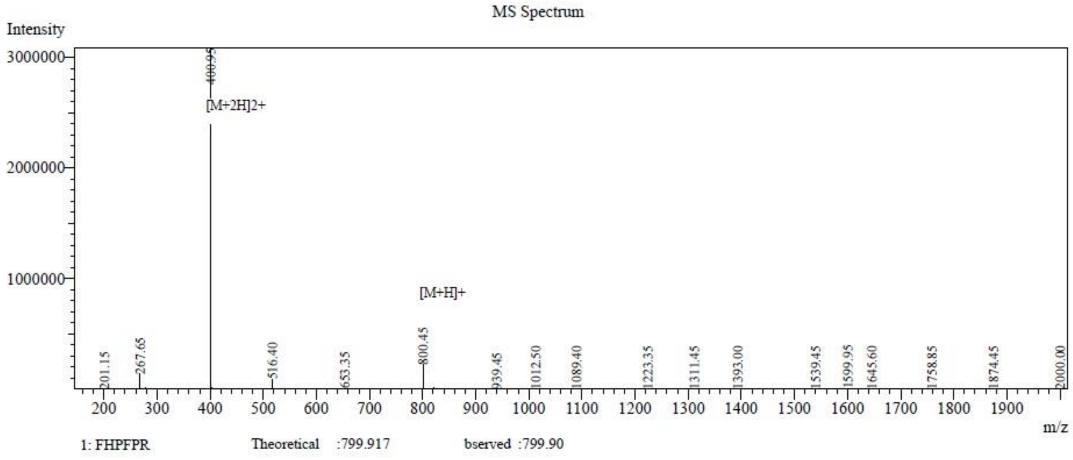

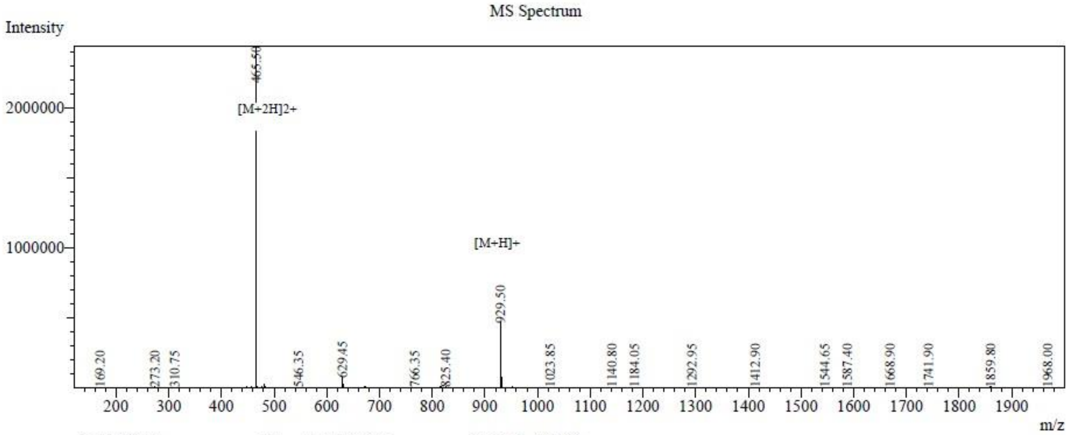

2: NWFPLPR Theoretical: 929.076 bserved :929.00

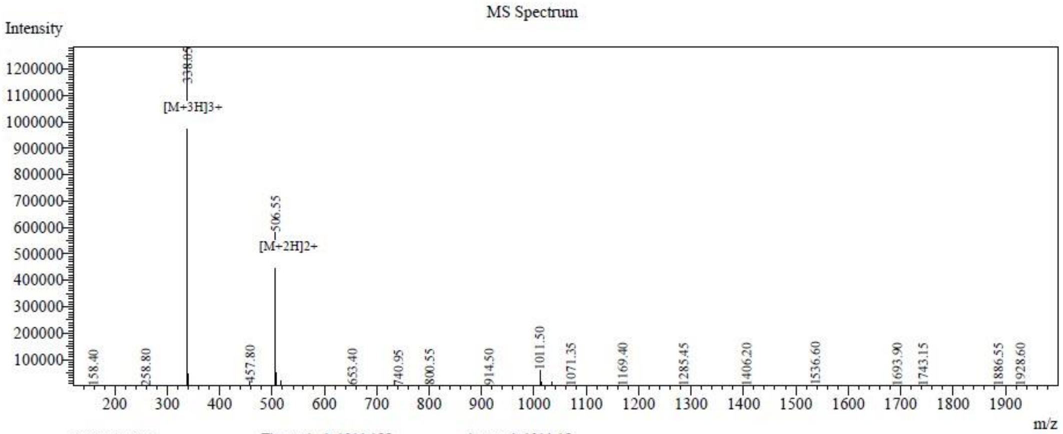

3: PNFHPFPR Theoretical: 1011.138 bserved: 1011.15

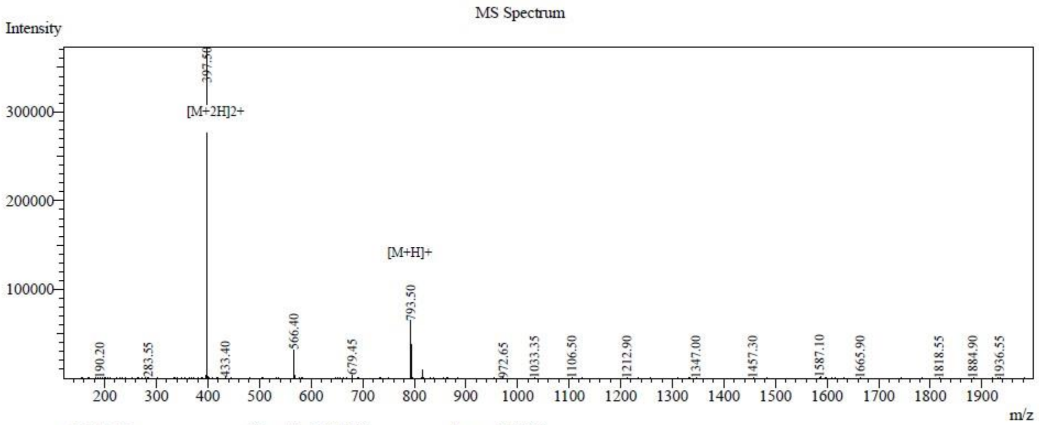

4: NIFRPF Theoretical: 792.923 bserved: 793.00

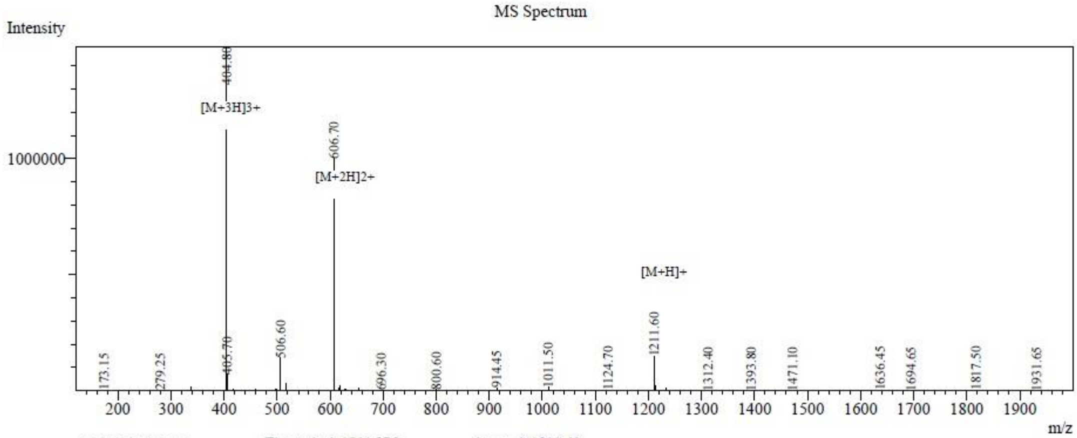

5: SLPNFHPFPR Theoretical: 1211.376 bserved: 1211.40

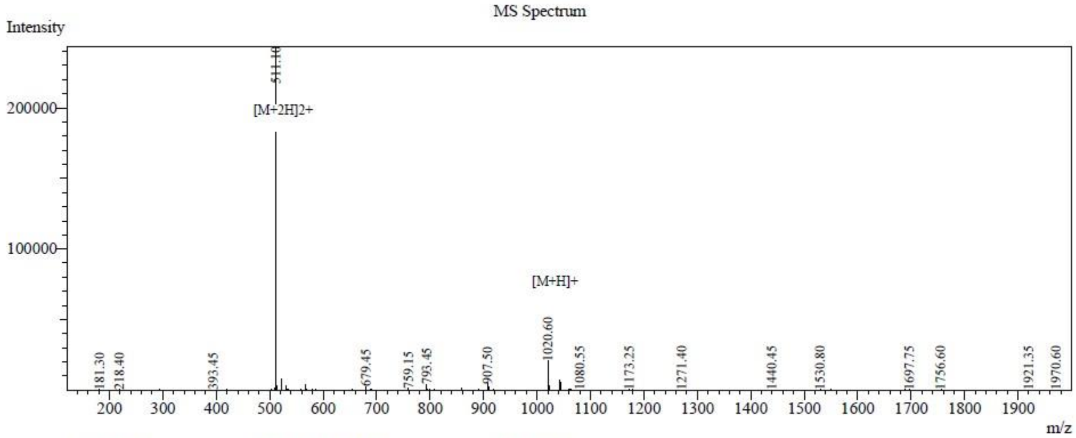

6: INNIFRPF Theoretical: 1020.187 bserved: 1020.20

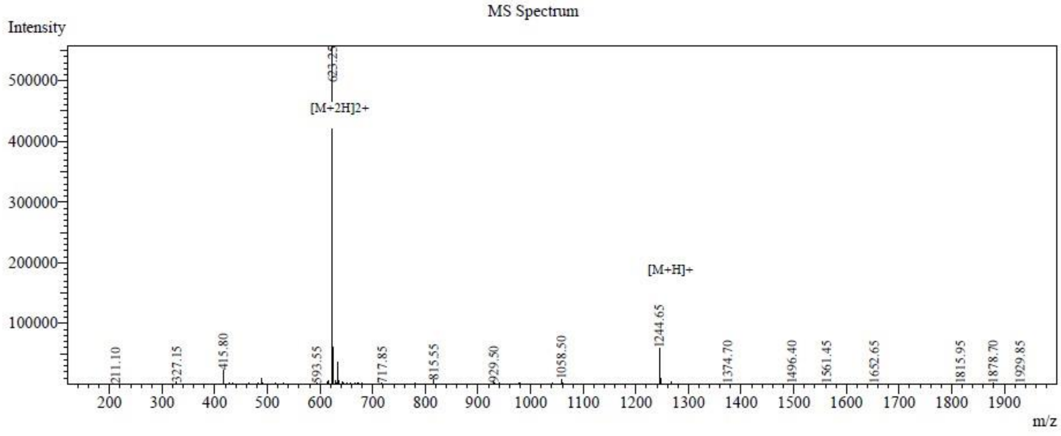

7: SVENWFPLPR Theoretical: 1244.403 bserved: 1244.50

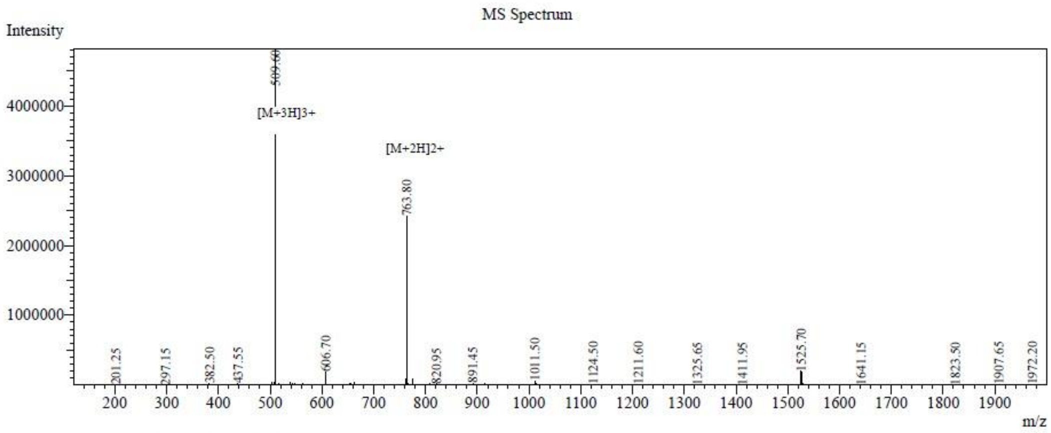

8: NSLSLPNFHPFPR Theoretical: 1525.718 bserve: 1525.80

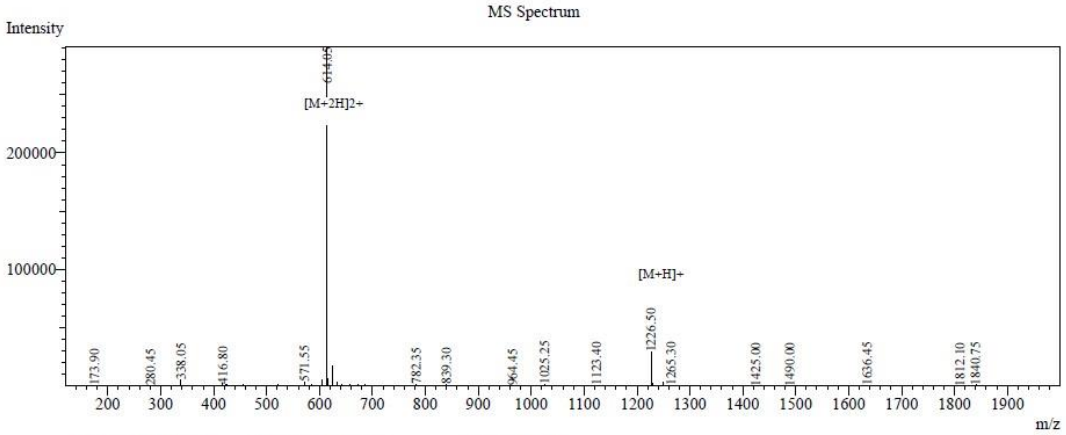

9: NSWGPNWGDHG Theoretical: 1226.215 bserved: 1226.10

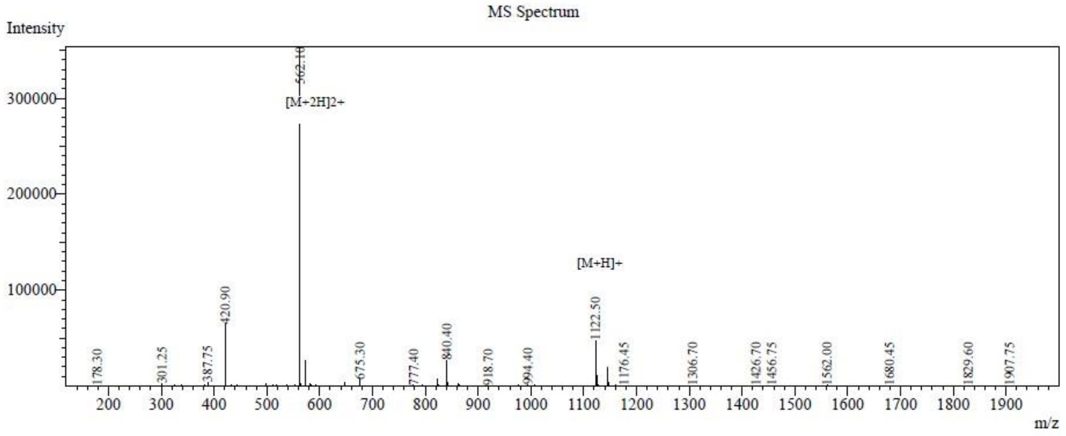

10: HYNPYFPGGA Theoretical: 1122.191 bserved: 1122.20

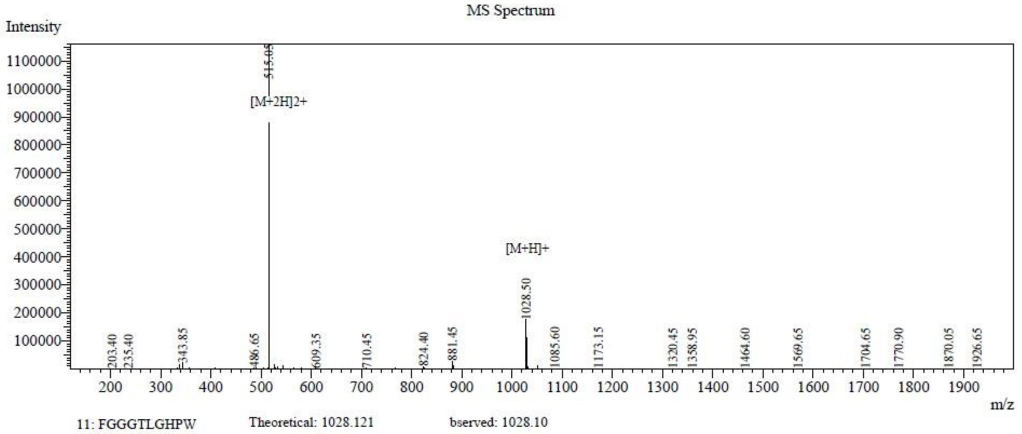

11: FGGGTLGHPW Theoretical: 1028.121 bserved: 1028.10

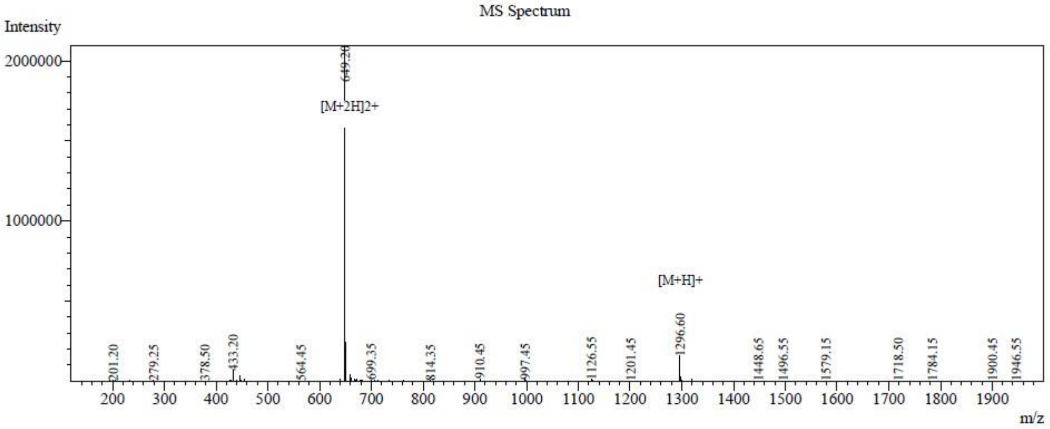

12: GLESPNYPWPH Theoretical: 1296.391 bserved: 1296.40

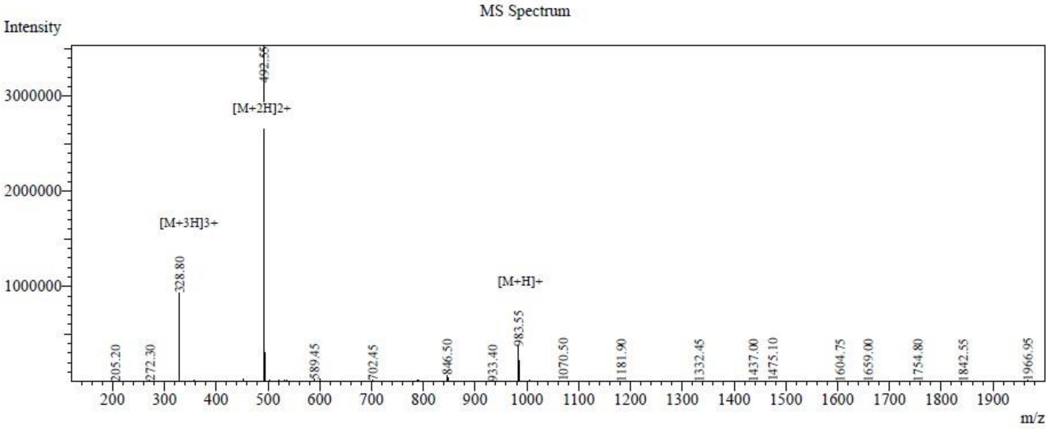

13: HGSLGFLPR Theoretical: 983.125 bserved: 983.10
